# Validation of an autonomous artificial intelligence–based diagnostic system for holistic maculopathy screening in a routine occupational health checkup context

**DOI:** 10.1007/s00417-022-05653-2

**Published:** 2022-05-14

**Authors:** Octavi Font, Jordina Torrents-Barrena, Dídac Royo, Sandra Banderas García, Javier Zarranz-Ventura, Anniken Bures, Cecilia Salinas, Miguel Ángel Zapata

**Affiliations:** 1 Optretina Image Reading Team, Barcelona, Spain; 2 BCN MedTech, Department of Information and Communication Technologies, Universitat Pompeu Fabra, Barcelona, Spain; 3 Facultat de Cirurgia i Ciències Morfològiques, Universitat Autònoma de Barcelona (UAB), Barcelona, Spain; 4 Ophthalmology Department Hospital Vall d’Hebron, Barcelona, Spain; 5 Institut Clinic of Ophthalmology (ICOF), Hospital Clinic, Barcelona, Spain; 6 Institut d’Investigacions Biomediques August Pi I Sunyer (IDIBAPS), Barcelona, Spain; 7 Instituto de Microcirugía Ocular (IMO), Barcelona, Spain

**Keywords:** Artificial intelligence, Screening, Retinography, Diabetic retinopathy, Age-related macular degeneration

## Abstract

**Purpose:**

This study aims to evaluate the ability of an autonomous artificial intelligence (AI) system for detection of the most common central retinal pathologies in fundus photography.

**Methods:**

Retrospective diagnostic test evaluation on a raw dataset of 5918 images (2839 individuals) evaluated with non-mydriatic cameras during routine occupational health checkups. Three camera models were employed: Optomed Aurora (field of view — FOV 50º, 88% of the dataset), ZEISS VISUSCOUT 100 (FOV 40º, 9%), and Optomed SmartScope M5 (FOV 40º, 3%). Image acquisition took 2 min per patient. Ground truth for each image of the dataset was determined by 2 masked retina specialists, and disagreements were resolved by a 3rd retina specialist. The specific pathologies considered for evaluation were “diabetic retinopathy” (DR), “Age-related macular degeneration” (AMD), “glaucomatous optic neuropathy” (GON), and “Nevus.” Images with maculopathy signs that did not match the described taxonomy were classified as “Other.”

**Results:**

The combination of algorithms to detect any abnormalities had an area under the curve (AUC) of 0.963 with a sensitivity of 92.9% and a specificity of 86.8%. The algorithms individually obtained are as follows: AMD AUC 0.980 (sensitivity 93.8%; specificity 95.7%), DR AUC 0.950 (sensitivity 81.1%; specificity 94.8%), GON AUC 0.889 (sensitivity 53.6% specificity 95.7%), Nevus AUC 0.931 (sensitivity 86.7%; specificity 90.7%).

**Conclusion:**

Our holistic AI approach reaches high diagnostic accuracy at simultaneous detection of DR, AMD, and Nevus. The integration of pathology-specific algorithms permits higher sensitivities with minimal impact on its specificity. It also reduces the risk of missing incidental findings. Deep learning may facilitate wider screenings of eye diseases.


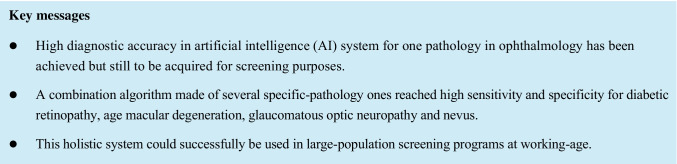


## Introduction

In 2010, 65% of those afflicted by blindness worldwide (32.4 million total) and 76% of those with moderate or severe vision impairment (191 million) had a preventable or treatable cause [1]. The causes vary among regions, but the trend since 1990 shows a decreased incidence due to cataract or refractive errors while age macular degeneration (AMD), glaucoma, and diabetic retinopathy (DR) are on the rise [1]. In developed countries, this trend is even more pronounced: AMD is the leading cause of blindness in people aged 75 years and older [2], whereas DR is the most frequent cause of preventable blindness in the working-age population (adults between 20 and 74 years old) [3]. Future projections do not show any sign of these diseases slowing down either. AMD will affect 288 million people in 2040 [4], glaucoma will impact 111.8 million in 2040 [5], and 191 million people will suffer DR by 2030 [6]. These diseases are treatable with good outcomes if detected early in the course of the disease [7–10], but they often are not symptomatic until late stages of their development. Thus, it is essential to have good screening systems for a timely diagnosis.

Running screening programs at large scale is costly, being the relatively high fixed costs per equipment the main driver of the charge [11]. This is even more noticeable in low-density areas, which are underserved by traditional screening approaches performed in primary-care settings [12]. Additional issues of such programs stem from their limited scope. Primary care physicians, with limited ophthalmological expertise, might often miss abnormalities outside the original screening program or have lower sensitivity than retinal experts [13, 14]. Ophthalmology is also a leading specialty in alternative forms of healthcare delivery. For instance, mobile digital non-mydriatic cameras are getting more affordable and have good specificity and sensitivity for DR [15, 16], which has enabled many screening plans in underserved areas [17–20]. There are also many examples of successful telemedicine screening plans in countries such as Australia, USA, India, Singapore, and Spain [21–24].

In parallel to the improvements in imaging and digitalization of healthcare, artificial intelligence (AI) based on deep learning (DL) [25] represents a breakthrough that has dramatically improved the state-of-the-art in many tasks such as speech recognition, image processing, and text generation, among others [26]. In the field of medicine, DL has been most successful in medical imaging analysis, by enabling the creation of computer-aided diagnosis systems (CADx) with expert-level accuracies. There are many examples in dermatology [27], radiology [28], gastroenterology [29], and ophthalmology [30, 31]. In fact, the first ever US Food and Drug Administration (FDA)-approved autonomous AI is a screening tool for DR [32].

While the results of the AI performance presented in many publications are encouraging, there are still questions to be answered regarding their real-world application. The vast majority of publications are limited to retrospective studies taken on datasets captured in populations with prior conditions in a hospital setting [33–35]. Also, most publications limit the scope of their algorithm to just one pathology which, while interesting, is not ideal for screening purposes [36].

This work aims to overcome both limitations, by presenting a retrospective study performed on 2839 patients evaluated by digital fundus images taken with handheld non-mydriatic cameras, on a routine checkup performed onsite at work centers. The algorithm evaluated in this study has previously been successful in detecting signs of DR, AMD, glaucoma, and nevus, the most common eye pathologies [24]. The novelty of our proposal is the combination of multiple pathology-specific algorithms to achieve holistic maculopathy detection. Each algorithm is trained to identify individual diseases, and, in conjunction, the final output increases the diagnostic accuracy of the AI system for ocular pathology detection.

## Materials and methods

### Study population

We present a retrospective diagnostic test evaluation. The dataset consists of 5918 images from a population of 2839 individuals, taken between the 9th of January and the 13th of March of 2020. The median age was 43 years old with a standard deviation of 11.52. From the study population, 1786 (63%) were male and 1053 female (37%) (see Table 1 for a detailed breakdown). Participants of this study were enrolled in a consecutive series during routine occupational health checkups offered by their employer as medical benefits [37]. All ophthalmologic check-ups were performed by a single provider (Optretina, Sant Cugat, Spain). The images were obtained by a trained technician using handheld non-mydriatic cameras on the participating center office premises, in a room which had been setup with adequate lighting conditions. The camera models employed were Optomed Aurora (field of view — FOV 50º, 88% of the dataset), ZEISS VISUSCOUT 100 (FOV 40º, 9% of the dataset), and Optomed SmartScope M5 (FOV 40º, 3% of the dataset). Image acquisition took around 2 min per patient. The raw image dataset was included in the study, and no images were discarded due to low resolution or were modified prior to the analysis.

**Table 1:** Breakdown of the study validation datasets, as well as the training datasets for each of the AI algorithms that compose the screening system. (*M/F* male/female, *LE/RE* left eye/right eye)

Dataset	*n* participants (M/F)	Age median (std) (range)	*n* eyes (LE/RE)	*n* images	Abnormal (%)	AMD (%)	DR (%)	GON (%)	Nevus (%)	Other (%)
Study validation										
Original	2839 (1786 M/1053 F)	43 (11.52) [5–87]	5483 (2747 LE/2736 RE)	5918	321 (5.42%)	14 (0.23%)	0 (0%)	107 (1.81%)	110 (1.86%)	90 (1.5%)
Original + enriched	3337 (1999 M/1338 F)	46 (15.22) [5–96]	6009 (3013 LE/2996 RE)	6452	855 (13.25%)	398 (6.17%)	150 (2.32%)	107 (1.66%)	110 (1.7%)	90 (1.4%)
Algorithm training										
AMD	– (982 M/1526 F)	75 (11.52) [5–98]	– (1761 LE/1945 RE)	7218	–	4859 (67.31%)	–	–	–	–
DR	71,455 (26,691 M/19,296 F)	66 (11.42) [11–99]	116,501 (60,947 LE/55,554 RE)	139,813	–	–	14,376 (10.28%)	–	–	–
Glaucoma	1206 (619 M/587 F)	54 (15.58) [5–96]	1738 (794 LE/944 RE)	2366	–	–	–	1168 (49.37%)	–	–
GON	18,750 (8.215 M/10,535 F)	42 (11.64) [5–96]	29,352 (15,633 LE/13,719 RE)	30,054	–	–	–	–	4470 (14.87%)	–
Abnormality	31,877 (13,843 M/18,034 F)	49 (17.33) [5–99]	52,791 (27,846 LE/24,945 RE)	53,194	17,433 (32.77%)	–	–	–	–	–

^a^The total number of participants as well as the total number of eyes for the AMD training dataset could not be obtained, since the non-pathological images used for training were not assigned to a patient. The reported numbers (M/F, LE/RE, age) are from the pathological cases, which were referenced to a patient

^b^The sex split and age median only accounts for 65% of the dataset. One of the image sources for this dataset did not include sex nor age information per patient

### Digital Fundus Image evaluation

**Fig. 1:**
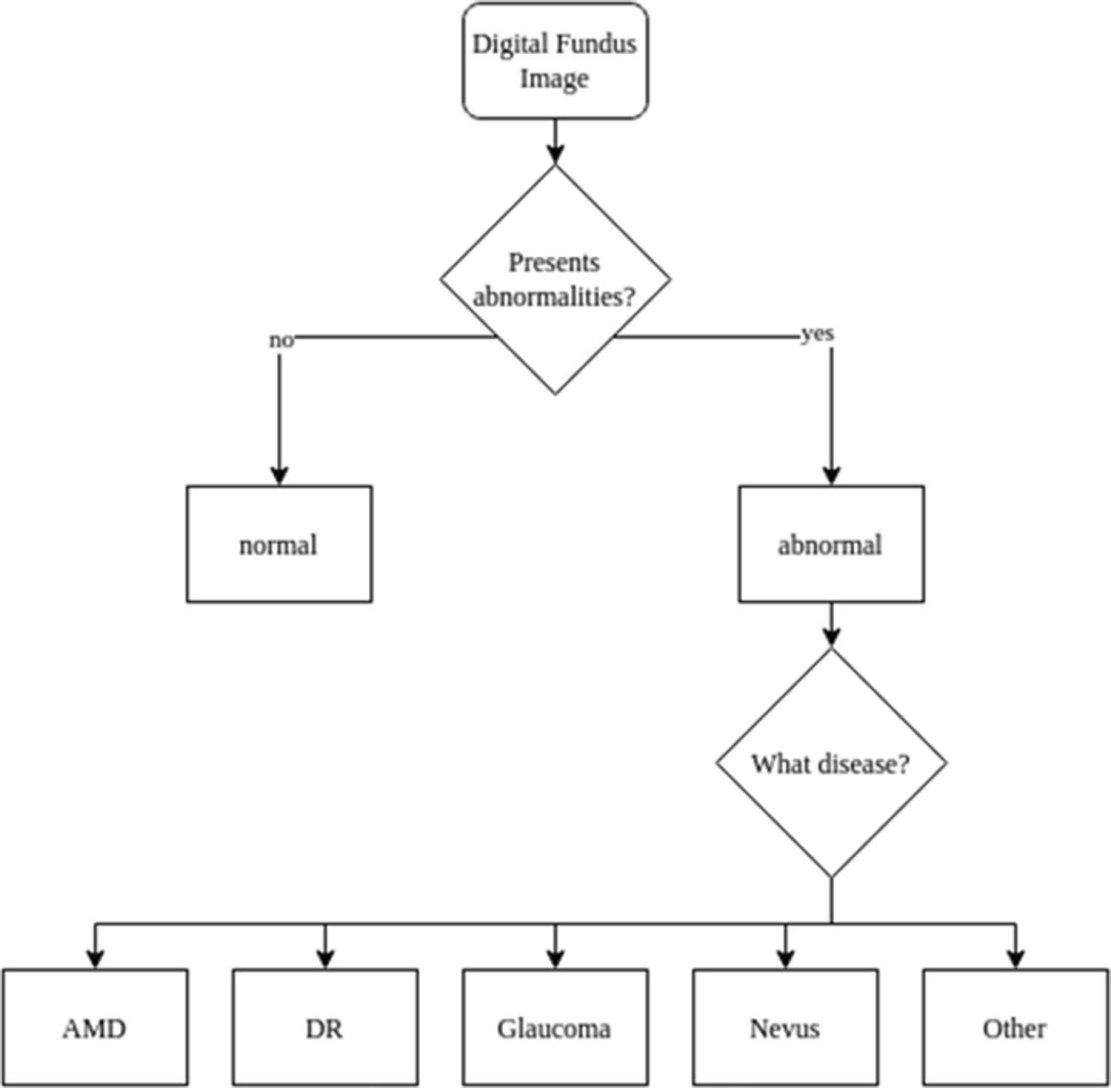
Labeling flowchart. The flowchart depicts the 2-tiered approach followed by all specialists to label the dataset. The ground truth was agreed by at least 2 graders

The ground truth of the data was evaluated per eye. For patients with multiple captures, an automated quality filtering was employed to select the highest-quality image. Afterward, each image was graded by 2 specialists (intragrader variability kappa of 0.86 and 0.79 respectively) in a 2-tiered approach (Fig. 1). In case of discrepancies, a 3rd retinal specialist reviewed the image (intragrader variability kappa of 0.83). The first step of the labeling process was to classify the image as “normal” or “abnormal,” considering the latter as any digital fundus image showing pathological signs. Abnormal images were further subclassified per pathology. The specific pathologies considered for evaluation were DR (defined as more than mild DR, as per the 2019 revision of the American Academy of Ophthalmology’s Preferred Practice Pattern) [38, 39], AMD (defined as mild or worse), GON (suspicious glaucomatous optic neuropathy was defined by a cup-to-disc ratio of 0.7 or more in the vertical axis and/or other typical changes caused by glaucoma, such as localized notches or retinal nerve fiber layer defects or peripapilar hemorrhages), and Nevus (defined with clinical parameters as an hyperpigmented lesion beneath the retina). Images classified as abnormal (with possible signs of maculopathy) not matching the described taxonomy were classified as other in tier 2.

### Dataset enrichment

We aim to assess the effectiveness of our automated screening algorithm on a wide-range general population. Because of the sampling bias of the initial population (working age participants, mostly without known prior pathologies), the prevalence for AMD and DR was far below that reported in the literature for the general population [4, 40]. To balance the data, we enriched the dataset with 384 AMD and 150 DR pathological images to match the prevalence in the general population of our environment [18, 41]. AMD images were obtained from Optretina’s image bank (the sample was randomly selected from a cohort of 2212 AMD cases screened from January 2013 to May 2020) [24]. DR images were randomly selected from a series of positive cases detected in the Institut Català de la Salut (ICS) screening program for diabetics (Barcelona, Spain). In both cases, the enriched images were labeled by two expert retinal specialists, following the procedure detailed in Fig. 1. The dataset details are shown in Table 1.

### Statistical analysis

The primary outcome of the analysis is the diagnostic accuracy of the AI system, defined by its sensitivity and specificity, versus the ground truth. Since the AI system performs a holistic screening, as well as pathology-specific diagnostic, we calculated the sensitivity and specificity for both. The operating threshold was fixed before the analysis and was not adjusted during the tests. The secondary outcomes are the receiver operating characteristic (ROC) curve and the area under the curve (AUC) index. All reported 95% CIs were obtained by performing a non-parametric bootstrap (1000 samples, with replacement). Study success was defined as reaching a predefined threshold of sensitivity and specificity on our holistic general screening algorithm. The hypotheses of interest were

$$H0:p<p0 vs HA:p\ge p0$$

where *p* is the sensitivity or specificity of the AI system. The predefined sensitivity and specificity thresholds were *p*0 = 0.75 and *p*0 = 0.775, respectively, benchmark defined by the FDA in their first-approved AI diagnostic system [32]. A one-sided 2.5% type I error binomial test was performed for both null hypotheses.

For the sample size calculation, we estimated a prevalence of retinal abnormalities in an occupational health checkup context of 7.8% with a 95% confidence interval, as per our previous study [42]. With these figures, the total number of participants needed was 2784. Additionally, we also confirmed that the sample size of our enriched dataset was large enough to ensure 80% statistical power (*β* = 0.2) on our sensitivity and specificity metrics, given the reported null hypothesis and the levels of pathological prevalence [43].

### Training dataset

For algorithm development, macula-centered digital fundus images were retrospectively obtained from Optretina’s own image bank (AMD, GON, Nevus, Abnormality) and Institut Català de la Salut and EyePacs (Kaggle). For AMD, Glaucoma, and DR, images were taken from a clinical setting, while Nevus and abnormality images were sourced from screenings, mostly performed with portable cameras. All images were evaluated by at least 1 expert retinologist, following the previously described criteria. The exact breakdown of the training dataset can be found in Table 1.

### Individual algorithms

For each dataset, we trained binary classificators (disease/no disease) using convolutional neural networks (CNNs). This process, with the right training data, allows the CNN to automatically learn features from the images that can be extrapolated successfully outside of the training data. The “AMD” algorithm uses a custom neural network architecture [42] using RGB images of 512 × 512 pixels [36]. The “DR” algorithm uses an InceptionV3 architecture [44] with inputs of 512 × 512.44, “Glaucoma” uses a ResNet50 [45] with inputs of 224 × 224.45, “Nevus” detection employs an InceptionV3 at 299 × 299, and the abnormal images detector another InceptionV3 at 299 × 299. The optimization algorithm to train the network was ADAM. We also used batch normalization, as well as using the weights of pretrained ImageNet networks where possible (InceptionV3, ResNet50) to speed up the training.

The performance of the algorithm was measured by the area under the receiver operating curve (AUC). The reported sensitivity and specificity points have been taken without adjusting the decision threshold (threshold = 0.5). The development datasets were split in an 80/10/10 fashion, where 80% of the data was used for training, 10% for validation (adjusting hyperparameters), and 10% to test the results. This data was split by patient (not image) and is completely independent from the dataset presented for the study validation.

### Screening algorithm

**Fig. 2:**
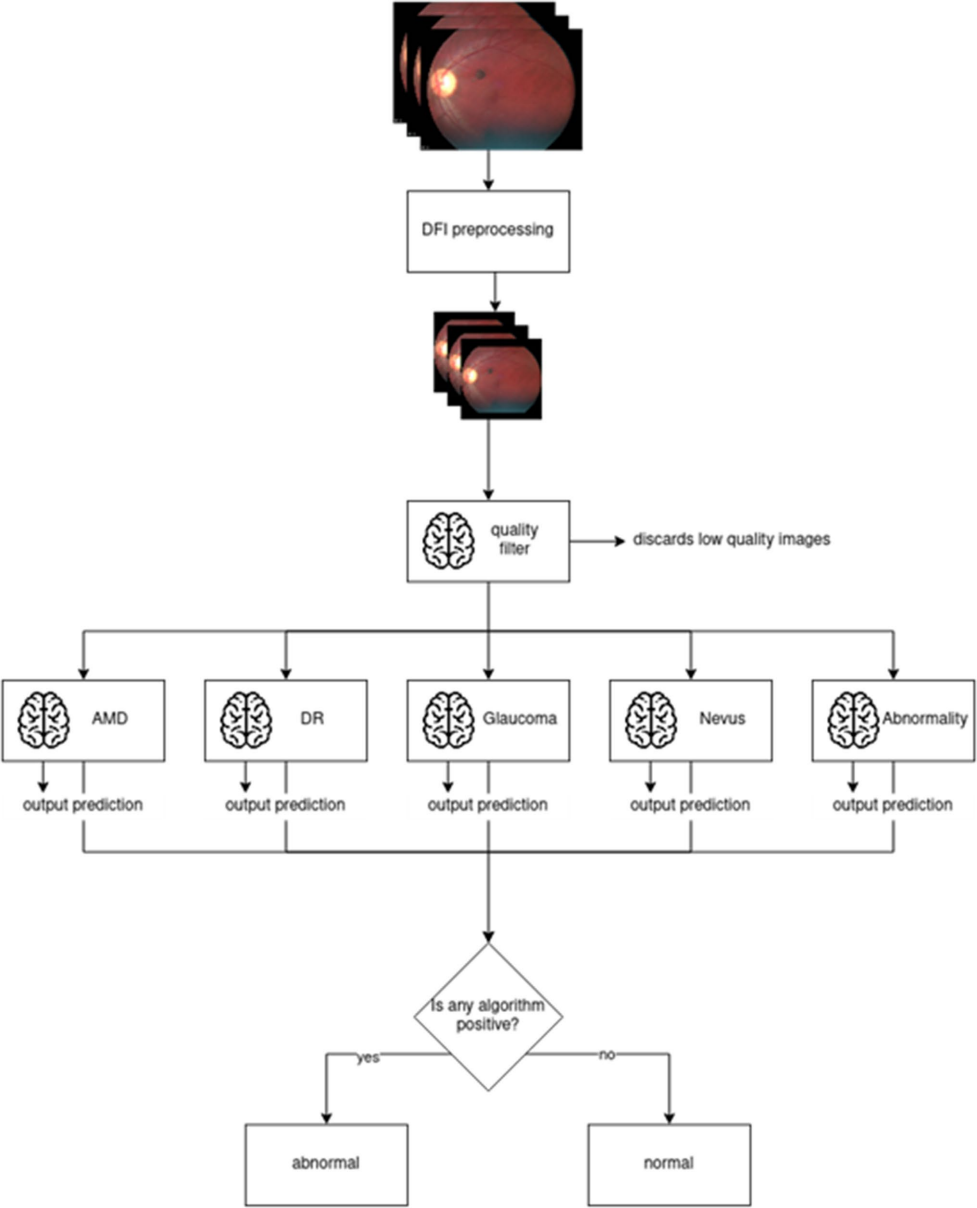
Algorithm execution flowchart. The predictions are performed at the image level. 5 neural networks process independently each image and in case any algorithm is positive, the screened image is classified as “abnormal”

The screening algorithm is a combination of five independently trained neural networks. Four of these neural networks target specific pathologies (AMD, DR, GON, and Nevus), while the fifth one has been trained as an outlier detector, with a training dataset containing images from the aforementioned pathologies as well as other undetermined maculopathies (Fig. 2). Each image evaluated by the system is processed independently by each of the five neural networks and, at a second step, their response is combined in a single output. If an algorithm detects signs of any of the individual pathologies, the screened image is classified as Abnormal. A complete diagram of the AI system architecture is presented in Fig. 2

## Results

The combined abnormality algorithm correctly identified 92% of the analyzable images annotated as Abnormal (776/843). The performance of individual disease algorithms is herein described: 99% of AMD images were correctly identified (394/398), 100% of DR images were correctly classified (150/150), 71% of GON images were correctly identified (71%, 75/107), 90% of Nevus images were correctly identified (99/110), and 73% of undetermined maculopathies were correctly classified (65/90). Insufficient quality of the images was observed in 0.23% of the cases (15/6452), which could not be graded. Of these, 80% were labeled as abnormal by our graders (12/15).

The single NN (neural network) abnormality algorithm correctly detected 82% (691/843) of the analyzable images. The percentage of images correctly classified disclosed by pathologies was 98.8% of AMD images (393/398), 94.6% of DR images (142/150), 45.6% of GON images (48/107), 49% of Nevus images (54/110), and 49% of other maculopathies images (44/90).

The AMD algorithm correctly detected 90% of AMD images (358/398). False positive rates were 62.6% of DR images (94/150), 6.7% of GON images (7/107), 0% of Nevus images (0/110), and 22.5% of other maculopathies images (20/90).

The DR algorithm correctly detected 68.6% DR images (103/150). False positive rates were 4.1% of AMD images (16/398), 0% of GON images (0/107), 0.9% of Nevus images (1/110), and 11.7% of other maculopathies images (10/90).

The GON algorithm correctly detected 58.2% of GON images (62/107). False positive rates were 10.6% of AMD images (42/398), 12.6% of DR images (19/150), 2.9% of 102 Nevus images (3/110), and 8.8% of other maculopathies images (7/90).

The Nevus algorithm correctly detected 88.2% of the Nevus-specific images (90/110). False positive rates were 88% of AMD images (350/398), 100% of DR images (150/150), 2.9% of GON images (3/107), and 63.7% of other maculopathies images (57/90).

Sensitivity and specificity obtained from all algorithms are summarized in Table 2. The threshold for each individual CNN was not adjusted to boost the sensitivity or specificity to a certain operating point. Sensitivity and specificity were calculated per eye, using the best quality image if multiple were available. Discarding eye duplicates had little effect in the metrics in our study cohort (sensitivity: 92.1% duplicates vs 92.8% non-duplicates; specificity: 87.6% duplicates vs 86.8% no duplicates). Enforcing high-quality standards in the preprocessing pipeline, the effect is more noticeable (sensitivity: 92.8% any quality vs 92.6% high quality; specificity: 86.8% any quality vs 89.1% high quality). The dataset, as classified by the automatic quality algorithm, consisted of 53.4% high-quality images (*n* = 3444), 43.6% of acceptable-quality images (*n* = 2809), and 3.0% of low-quality images (*n* = 196).

**Table 2:** Summary of sensitivity, specificity, and AUC aggregated and per individual algorithm

Algorithm	Sensitivity (95% CI) [*p*-value]	Specificity (95% CI) [*p*-value]
Combined algorithm:		
Abnormality	92.9% (91.0, 94.6) [< 0.001]^a^	86.8% (85.8, 87.7) [< 0.001]^b^
Single NN algorithms:		
Abnormality	83.4% (80.6, 85.9)	93.4% (92.7, 94.0)
AMD	93.8% (91.6, 96.3)	95.7% (95.2, 96.2)
DR	81.1% (75.3, 88.1)	94.8% (94.1, 95.4)
Glaucoma	53.6% (43.1, 63.1)	95.7% (95.2, 96.2)
Nevus	86.7% (80.7, 94.0)	90.7% (90.1, 91.5)

^a^*p*-value for sensitivity on the combined abnormality algorithm was computed using a one-sided tailed binomial test using a sensitivity of *p* = 0.75 as the null hypothesis

^b^*p*-value for specificity on the combined abnormality algorithm was computed using a one-sided tailed binomial test using a sensitivity of *p* = 0.775 as the null hypothesis

**Fig. 3:**
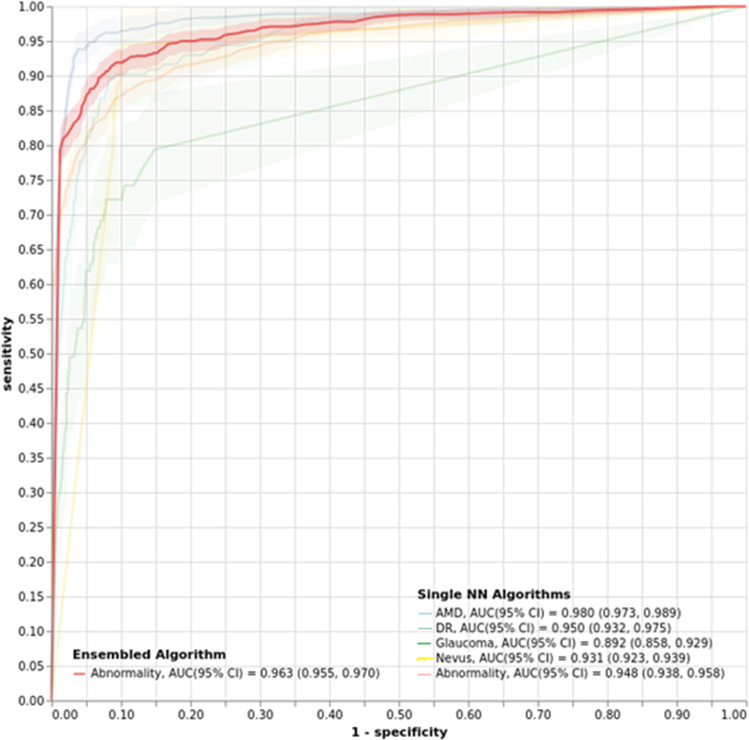
Receiver operating curve for the combined and individual algorithms

To represent the best operation points, we plotted the ROC curve for the combined algorithm and the individual models in Fig. 3. Additionally, we also computed the AUC of the combined algorithm (0.963) and the single NN version (0.948).

## Discussion

The proposed study has exceeded the expectations of the holistic solution proposed for screening the central retina’s diseases. The reported diagnostic accuracy levels are similar to other algorithms already available on the market and higher than those required by the FDA for approval [32]. However, the proposed system presents the additional benefit that several pathologies are simultaneously screened together with those that cause greater visual loss among industrialized countries. These are also the main causes of preventable, non-reversible blindness, which are experiencing more growth in the world [46].

It is widely demonstrated that early detection of these diseases (mainly AMD, DR, and GON) and their early treatment, if necessary, can prevent visual loss in a very high percentage of patients [47–49]. Simultaneous screening for multiple pathologies of the retina has previously been contemplated in some publications, both associated with human reading and artificial intelligence, mainly combining the detection of AMD and DR [14, 50], and also glaucoma [51]. To date, all artificial intelligence studies using fundus images for other diseases than diabetes have been carried out only in existing databases, with no clinical validation studies performed prospectively.

Currently, screening programs in most countries focus on DR, probably for cost-effectiveness reasons. Our study population is relatively young, and a priori, healthy, despite previous studies that report alterations in fundus images in almost 8% of cases in this type of population [37]. Although these alterations do not usually represent serious or urgent cases, any pathology in this population, young and working, can have significant socio-economic repercussions. The incorporation of artificial intelligence and the simultaneous screening of several diseases can make these early detection systems more cost-effective. Despite the fact that the objective of this study is not an economic evaluation, the use of automatic detection software can reduce previously reported costs, lower than 10 euros per patient [37].

One of the most important causes of loss of effectiveness of AI is related to the quality of the images [52]. In our series, we have had 3% of fundus photographs of low quality, a figure significantly lower than other published series such as Abramoff et al. (above 8%) [32] or Liu et al. (16%) [53]. We would like to note that our numbers are obtained on real conditions, with portable cameras and, generally, under certainly strict timeframes. This difference can be due to different reasons. While portable cameras traditionally tended to be of lower quality than desktop cameras, the technical advances in recent years have improved the quality of the images, and current cameras like Aurora are of equivalent quality. Moreover, screening has been carried out in relatively young people, in which ocular media opacities are much less common and tend to have more dilated pupils in scotopic conditions. One of the most interesting points is the study of comorbidities; to date, it is also one of the limits set by AI. With this type of approach, lesions can be detected by independent algorithms that combine together in a holistic diagnosis. This results in a more robust system, less likely to miss incidental findings, with higher overall sensitivity, while only penalizing slightly on specificity. The use of AI for DR screening is already being implemented successfully in some countries. However, this pathology-specific approach carries the risk of ignoring other possible findings, since the neural networks employed are not designed for it. We do believe that it would be beneficial if those IA systems are combined and set up with a more holistic approach, to minimize the risk of ignoring these incidental findings. The combination of multiple algorithms also makes it easier to deploy improvements on the system. We can tackle algorithms per pathology, and any improvements in the individual models will benefit in the final output. We have already achieved gains in multiple retrainings of the DR algorithm, and we believe that the rest of algorithms could be similarly improved in the near future. GON algorithm alone shows weaker performance than the rest of single algorithms, making the single and combined algorithms not reliable for screening of this pathology.

This study’s limits are those determined by carrying out a retrospective study, those related to the population studied (in this case, younger and with a lower rate of pathology than the general population), and the limits derived from the pathology studied. To compensate for possible biases, the database has been enriched with a presence similar to the population of AMD and DR. It would be convenient, in the future, to introduce other highly prevalent pathologies in the population, such as the presence of epiretinal membranes or macular signs associated with high myopia. Another area that we want to study further is in the image capture workflow, to offer not only an automated way of screening, but a better screening workflow with hybrid systems. We believe that by integrating the image acquisition process with an online platform for automated data collection, it is possible to instrument the whole process and guide the technician through, with the additional benefit that the images are automatically assigned to the right patient and checked for adequate quality prior to running any subsequent diagnostic analysis.

In conclusion, the use of an autonomous AI-based diagnostic system based on fundus images for holistic maculopathy screening in a routine occupational health checkup context seems effective, with high levels of sensitivity and specificity that improves further those achieved by specific algorithms. The application of these systems could allow more extensive screening programs with greater detection of pathology in working-age patients.

## Data Availability

Any information not included in the present manuscript is available upon request by contacting the corresponding author.

## Code availability

The AI system described in this study is copyrighted by Optretina S.L. and consequently the underlying source code is not available.
